# Metabolic Endotoxemia: A Potential Underlying Mechanism of the Relationship between Dietary Fat Intake and Risk for Cognitive Impairments in Humans?

**DOI:** 10.3390/nu11081887

**Published:** 2019-08-13

**Authors:** Perrine André, Fabienne Laugerette, Catherine Féart

**Affiliations:** 1 Team Lifelong Exposure Health and Aging, Inserm, Bordeaux Population Health Research Center, Univ. Bordeaux U1219, F-33000 Bordeaux, France; 2 Univ-Lyon, CarMeN Laboratory, INRA U1397, Inserm U1060, Université Claude Bernard Lyon 1, INSA Lyon, Charles Mérieux Medical School, FR-69600 Oullins, France

**Keywords:** nutrition, dietary fat, high-fat, endotoxemia, lipopolysaccharide, Alzheimer’s disease, dementia, humans

## Abstract

(1) Background: Nutrition is a major lifestyle factor that can prevent the risk of cognitive impairment and dementia. Diet-induced metabolic endotoxemia has been proposed as a major root cause of inflammation and these pathways emerge as detrimental factors of healthy ageing. The aim of this paper was to update research focusing on the relationship between a fat-rich diet and endotoxemia, and to discuss the potential role of endotoxemia in cognitive performances. (2) Methods: We conducted a non-systematic literature review based on the PubMed database related to fat-rich meals, metabolic endotoxemia and cognitive disorders including dementia in humans. A total of 40 articles out of 942 in the first screening met the inclusion criteria. (3) Results: Evidence suggested that a fat-rich diet, depending on its quality, quantity and concomitant healthy food components, could influence metabolic endotoxemia. Since only heterogeneous cross-sectional studies are available, it remains unclear to what extent endotoxemia could be associated or not with cognitive disorders and dementia. (4) Conclusions: A fat-rich diet has the capability to provide significant increases in circulating endotoxins, which highlights nutritional strategies as a promising area for future research on inflammatory-associated diseases. The role of endotoxemia in cognitive disorders and dementia remains unclear and deserves further investigation.

## 1. Introduction

As a consequence of the ageing population, the prevalence of dementia, characterized by a progressive deterioration of cognitive performances in multiple domains (i.e., memory, reasoning, judgement and ability to perform daily activities) that evolves into a pathological diagnosis, is increasing [1]. The aetiology of dementia is multi-factorial and consists of a dynamic interaction between genetic susceptibility, non-modifiable factors (i.e., age and sex), pathological processes and environmental factors, some of them being potentially preventable [2,3].

In this context, nutrition—a major lifelong environmental factor—is of growing interest and offers an interesting strategy for the prevention of cognitive decline and subsequent dementia [4]. Numerous studies have suggested a benefit of higher adherence to healthy dietary patterns, with a beneficial balance in favour of unsaturated fatty acids to the detriment of saturated fatty acids such as the plant-based Mediterranean diet, on age-related cognitive impairments, whether they are due to degenerative or vascular origin [5,6,7,8].

Conversely, the Western diet, characterized among other factors by a high intake of saturated and trans fatty acids, has been associated with an increased risk of developing dementia [5,7]. Moreover, adopting this fat-rich dietary pattern can lead to metabolic disorders such as obesity or Type 2 Diabetes Mellitus (T2DM), these latter being associated with a higher risk of developing dementia in later life [9,10].

Nutritional strategies represent a promising area to prevent neurocognitive impairments and subsequent dementia. While the precise mechanisms underlying the relationships between these dietary habits on age-related cognitive disorders are not yet completely understood (for instance, inflammatory pathways, vascular factors, oxidative stress or amyloidogenesis [11,12,13,14]), we suggest that the activation of the innate immune system by endotoxins—which are still poorly described—could be considered as one potential cellular mechanism involved in these relationships.

Endotoxins (Lipopolysaccharides, LPS), a major component of the outer membrane of Gram-negative bacteria, are now well as contributors to the inflammation [15]. Although LPS may originate from skin and mucous membranes or local sites of bacterial infection for instance, the gut microbiota is considered the main natural reservoir of pro-inflammatory endotoxins in the body [16]. Endotoxins are released when bacteria die, and then dissociated endotoxins are able to cross the gastro-intestinal barrier to end up in the bloodstream. The presence of LPS in the bloodstream is defined as endotoxemia. Briefly, the endotoxic metabolic pathway includes the binding of circulating LPS to LPS-Binding Protein (LBP) and its transfer to the CD14 receptor, which is present both in a membrane-anchored form (mCD14) and in a soluble circulating form (sCD14) [17]. The complex LPS–LBP–CD14 initiates the secretion of pro-inflammatory cytokines, such as Interleukin-6 (IL-6) or Tumor Necrosis Factor α (TNFα), through a TLR4-dependent mechanism [18].

Although LPS is detectable at low concentrations in the circulation of healthy individuals, there is evidence that LPS levels transiently increase following ingestion of fat-rich meals [19]. Such endotoxemia is defined as “metabolic endotoxemia”, in contrast to other sources of endotoxemia such as exogenous bacterial infection or sepsis. For instance, in mice fed with a four-week high-fat diet, plasma levels of LPS were similar to those observed following a four-week subcutaneous infusion of 300 µg/kg/day of LPS [20]. Metabolic endotoxemia has been proposed as a major cause of inflammation, including chronic low-grade inflammation. Indeed, animal and experimental studies have demonstrated that postprandial state may result in an inflammatory response closely associated with the increase in the circulating levels of LPS [21,22]. Interestingly, not all dietary interventions lead to increased metabolic endotoxemia and the associated postprandial inflammatory status. Data from animal studies have suggested that dietary fats act as the main macronutrients responsible for postprandial endotoxemia, and that both the quantity and the quality of the dietary fat differentially influence metabolic endotoxemia [23,24,25]. Moreover, healthy diets rich in unsaturated fatty acids have been associated with lower postprandial circulating levels of LPS, closely associated with lower pro-inflammatory markers. Conversely, the consumption of high-energy or high saturated-fat diets has been associated with increased postprandial levels of LPS and increased circulating levels of pro-inflammatory markers [23,24,25].

Currently, the available literature points out that elevated circulating levels of LPS are detrimental for healthy ageing. More specifically, elevated levels of LPS have been associated with a large range of diseases such as obesity, T2DM, coronary artery disease and depression [19]. In the context of dementia, studies have summarized evidence that neuroinflammation is considered not only an epiphenomenon of Alzheimer’s disease (AD) but also a triggering factor that contributes to exacerbated AD pathology [26,27]. In addition, systemic exposure to an overload of endotoxins is widely used in animal studies to induce neuroinflammation and neurodegeneration and is associated with increased disruption of the amyloid-β precursor, hyperphosphorylated tau and neuronal cell death, which are hallmarks of AD [15,28,29,30,31]. Based on the biological plausibility taken from animal studies, we hypothesized that lifelong exposure to endotoxins, worsened by a chronic consumption of unhealthy diets rich in saturated fat, might exacerbate the detrimental outcomes of endotoxins in the human brain. The aim of this paper is therefore to discuss the potential role of endotoxins, as an underappreciated mechanism that could be involved, at least in part, in the relationship between dietary fat intake and the pathogenesis of dementia in humans.

This article was thus conducted to emphasize research that focused on two major approaches: (i) to determine the impact of fat-rich meal intake on postprandial and fasting endotoxemia and then (ii) to clarify the relationship between LPS and cognitive performances, with a specific emphasis on dementia or AD wherever possible.

## 2. Methods

We did not follow the strict methodology described for conducting a systematic literature review; since not all selected studies were reviewed by the three co-authors, there was no standardized quality assessment of selected studies and we restricted our research to a single database (i.e., PubMed). The literature search was conducted and closed in February 2019 to encompass the two main aspects described above.

We used the following nutritional search terms: “high-fat”, “fat intake”, “dietary fat”, “(un)saturated fatty acids”, “Western diet”, “(un)healthy diet”, “Mediterranean diet”, “prudent diet” combined with at least one of the search terms relative to LPS, which included “lipopolysaccharide”, “LPS”, “endotoxin”, “endotoxemia”. These search terms were applied for the selection of articles related to the approach focusing on the relation between dietary fat intake and metabolic endotoxemia. Regarding the approach focusing on the relation between endotoxemia and cognitive performances/dementia, identical search terms have been applied for LPS, combined with at least one of the following cognition-related terms: “dementia”, “Alzheimer’s disease”, “cognition”, “memory”, “neurocognitive disorders”. A literature search was also applied with the combination of all three domain terms (i.e., nutritional AND LPS AND cognitive terms) to select any article that would concern the whole approach. Other studies were obtained manually from the reference lists of review or original articles.

Through this research strategy, we identified a total of 942 original articles conducted on human samples (299 related to the relation between dietary fat intake and metabolic endotoxemia and 643 related to the relation between endotoxemia and cognitive performances/dementia).

Article selection was based on a two-step procedure with a first step selection based on the title and a second step selection based on the abstract reading. Articles were selected applying the following inclusion criteria: articles published in English, in scientific journals, original research, conducted on human samples. Articles including animal studies, in vitro and ex vivo studies, studies on vaccination with LPS or other pre-treatment with LPS, population aged less than 18 y, systematic review and meta-analyses were excluded. No restriction was performed on study design or date of publication.

Due to the possible sickness behavior induced by endotoxin injection (e.g., fever, malaise, headache and so forth) which may influence the subjective aspect of well-being and cognitive feeling, we chose not to discuss studies about self-reported feelings of the cognitive aspect or mood disorders. Finally, a total of 40 articles were included to establish this “state-of-the-art” article.

Due to the heterogeneity observed in nutritional intervention studies and regarding the cognitive domains assessed, no meta-analyses were performed in this article.

## 3. Results

### 3.1. Dietary Fat and Endotoxemia

#### 3.1.1. Role of Dietary Fat Interventions on Postprandial Metabolic Endotoxemia

The role of dietary fats is of growing interest among macronutrients in many studies on the link between nutrition and metabolic endotoxemia. As described below (Table 1), evidence accumulated over the past few decades has demonstrated a transient increase in circulating LPS following the consumption of dietary fats.

##### Dietary Fats

Deopurkar et al. [32] attempted to determine which macronutrient was responsible for postprandial metabolic endotoxemia by comparing isocaloric meals (300 kcal) from a glucose-drink, orange juice or dairy cream among 42 healthy adults. In this study, only dairy cream consumption increased plasma LPS levels, suggesting that dietary fats act as the main macronutrient responsible for postprandial endotoxemia. Among healthy men, postprandial LPS was increased by 35% to 50% following a single acute high-fat bolus [21,33,34].

##### Amount of Fats

In a comparative study using different caloric doses of a high-fat diet (500, 1000 or 1500 kcal corresponding to 34 to 102 g of fat), Schwander et al. [35] observed a positive association between fat caloric doses and increased LPS levels. Along this line, another study found higher postprandial levels of LPS following ingestion of 40 g of fat compared to 10 g among obese participants [36].

##### Quality of Fats

More recently, a study suggested that not only the quantity but also the quality of dietary fats may be of importance. In a randomized crossover study among 20 healthy young adults, Lyte et al. [37] demonstrated that serum levels of LPS were decreased by 50% following the consumption n-3 polyunsaturated fatty acids (PUFA) and increased by 60% following the consumption of saturated fatty acids (SFA). A sustained response (until 3 h after meal) was also observed in high-fat diet compared to low-fat-diet (peak at 1 h after meal).

##### Structure of Fats

Interestingly, beyond the quantity and quality of fat intake, Vors et al. [38] suggested that the structure of lipids could also be one of the determinants of metabolic endotoxemia and compared the effect of 40 g of milk fat, which only differed by fat structure, either spread or emulsified. Compared with spread fat consumption, emulsified fat consumption induced more pronounced postprandial levels of LPS in obese subjects.

##### Supplementation with Healthy Food Components

Acute peanut consumption within a high-fat meal was associated with lower LPS concentrations 3 h postprandially compared with a control biscuit which provided similar quantities of macronutrients, fibre and energy [39]. This result suggested that the addition of healthy food components to a high-fat diet could contribute to limiting postprandial metabolic endotoxemia. In this line, Ghanim et al. demonstrated that the increased levels of LPS following the intake of a high-fat high-calorie meal [40] could be prevented by the addition of 30 g of fibre, among healthy adults [41]. In parallel, no increase in postprandial endotoxemia was observed after the consumption of a low-fat meal rich in fibre and fruit [40]. Using the same high-fat high-calorie meal as Ghanim et al. [40], Milan et al. [42] explored the postprandial effect of dietary fat intake among healthy younger (20–25 y) and older adults (60–75 y) and failed to reproduce the postprandial effect observed by Ghanim et al., although a trend for higher LPS levels was observed with the high-fat high caloric diet. Moreover, no difference in LPS levels was observed between younger and older adults following meal intake.

Overall, these studies suggested that the matrix effect of foods (i.e., the quantity, the quality, and structure of the ingested fats and the overall food components of the diet) could be a key component of the diet intake-related metabolic endotoxemia response, although no report has specifically developed this hypothesis.

##### Metabolic Endotoxemia among Different Disease Phenotypes

The acute effects of dietary fats intake on metabolic endotoxemia among different disease phenotypes including obesity, impaired glucose tolerance (IGT) and T2DM have also been explored. Interestingly, the overall results suggested that metabolic endotoxemia was significantly associated with metabolic health as described below.

In 2012, Harte et al. [43] found that metabolic endotoxemia was exacerbated after dietary fat intake among individuals with IGT and T2DM compared with non-obese participants. Following the ingestion of whipping cream, postprandial endotoxin levels were increased by 20% in obese or IGT subjects and by 125% in T2DM individuals compared with non-obese subjects. Among 40 morbidly obese adults (i.e., BMI > 40 kg/m²), the ingestion of a fat overload increased levels of LPS in participants with the highest postprandial hypertriglyceridemia (>80 mg/dL) [44]. More recently, Al-Disi et al. [45] demonstrated that a high-fat diet (75 g of fat per m² body surface) exacerbated the postprandial endotoxemia in normal-weight and T2DM participants, but with a different impact on cardio-metabolic health: non-diabetic subjects appeared to have better metabolic resistance to the insult of a high-fat diet as seen by a postprandial increase in triglycerides and insulin and a decrease in HDL and LDL cholesterol [45].

Overall, evidence suggests that a single high-fat meal, especially one devoid of healthy food components, has an undoubted capacity to enhance transient postprandial endotoxemia in humans, this latter finding appears to be more pronounced in metabolically impaired individuals. To date, only a few studies have investigated the long-term effect of diet on postprandial and fasting levels of LPS, as described below.

#### 3.1.2. Associations between Long-Term Dietary Interventions and Circulating LPS

In an intervention study, the increase in energy intake (+70 g fat for eight weeks) was associated with an acute rise in endotoxin levels in the postprandial state, but not in the fasting state [46] (Table 2). More recently, three weeks of an intervention based on a low-fat high-carbohydrate diet enriched in n-3 PUFA has been shown to increase the postprandial levels of LPS, but decrease the fasting levels of LPS, compared with a Mediterranean diet enriched in MUFA or a SFA-rich diet, among healthy older subjects [47]. Among 75 metabolically impaired subjects, adherence to a high-fat high-saturated-fatty acid diet for 12 weeks led to an increase in the postprandial levels of LPS, but not the fasting levels [48].

In a crossover study among eight healthy older adults (55 y and over), Pendyala et al. [49] demonstrated that following a Western-type diet for four weeks significantly increased fasting plasma levels of LPS by 71%. In contrast, a 38% decrease in fasting plasma LPS levels was observed after four weeks of adherence to a prudent-type diet with equivalent energy intake to the Western-type diet. Regardless of the source of energy, Breusing et al. [50] observed that the 31% increase in fasting endotoxemia after one week of overfeeding (+50% of the energy requirement) was reversed by three weeks of caloric restriction (−50% of the energy requirement) among 15 healthy adults.

#### 3.1.3. Associations between Lifestyle Dietary Patterns and Circulating LPS (Table 2)

Although of major interest, only very few studies have investigated the relationship between usual diet and circulating levels of LPS, all using a cross-sectional design analysis and exhibiting inconsistent results. In a subsample of 201 healthy men, fat and energy, but not carbohydrate or protein, intake was positively associated with fasting levels of LPS [51]. More recently, Kallio et al. [52] reported an association between energy intake and the levels of LPS observed only among lean subjects. Surprisingly, no association between fat intake and the fasting levels of LPS was observed in this study. On the other hand, two studies did not find an association between nutrient intake and fasting LPS levels among overweight and obese pregnant women [53] or type 1 diabetes patients [54]. In this latter study [54], higher consumption of fish and healthy snacks (including fruits and berries, fresh vegetables, soft drinks, yoghurt and low-fat cheese) and higher adherence to a modern diet (composed of fresh vegetables, pasta and rice, poultry, meat dishes and fried or grilled foods) were all significantly associated with lower fasting LPS levels in serum [54]. Finally, among elderly patients with nonvalvular atrial fibrillation, higher adherence to a Mediterranean-type diet was inversely correlated with fasting circulating LPS levels [55]. Interestingly, among Mediterranean diet food components, higher intake of fruits and legumes showed a major association with lower levels of LPS.

Overall, these results underlined that a transient rise in circulating levels of LPS can be induced by a large variety of high-fat diets, especially those devoid of healthy food components. Moreover, fasting or postprandial assessment of LPS could also partly explain the discrepancies observed in several studies. Considering that exposure to an overload of endotoxins could contribute to the development of adverse health outcomes such as elevated systemic inflammation, neuroinflammation, neurodegeneration and neural death in experimental studies [15,56], we hypothesized that lifelong exposure to endotoxins inherent to adherence to unhealthy diets and to ageing could worsen the detrimental outcomes of endotoxemia. The second part of this state-of-the-art paper describes the few studies focusing on the relationship between endotoxemia and cognitive disorders and dementia in humans.

### 3.2. LPS, Cognitive Disorders and Dementia

#### 3.2.1. LPS Injection and Short-Term Cognitive Function Assessment in Interventional Studies

In the field of LPS-related inflammation in association with dementia or cognitive performances, few studies have been identified following our research strategy.

First, all studies were interested in the impact of LPS injection on the inflammatory response of the host. Transient increases in pro-inflammatory cytokines (for instance IL-6 and TNFα, on average between 1 and 6 h post-infection) followed by the release of anti-inflammatory cytokines (for instance IL1-Ra and IL-10, on average between 3 and 8 h post-infection), were observed after LPS injection in all intervention studies. This result was consistent regardless of the LPS dose injected. Second, several lines of evidence suggested that inflammatory cytokines such as IL-6 or TNFα could be involved in cognitive disturbances [26,27]. However, it remains unknown whether and to what extent cognitive functions could be affected during transient immune activation induced by a single injection of LPS, as provided by the selected intervention studies described below (Table 3).

In a crossover study, Reichenberg et al. [57] reported a significant impairment in declarative memory until 10 h after the injection of LPS. With this intervention, decreased performances in declarative memory have also been observed in a subsample of subjects, as well as improvements in working memory compared to the injection of placebo [58]. These last controversial results are, however, not generalizable to the whole study sample [57], which raises questions. Finally, in both studies [57,58], there were no statistical or clinical differences regarding attention or executive functions following the injection of LPS compared to placebo.

In another study, using an injection of a lower dose of LPS (0.2 ng/kg body weight) a negative correlation was found between the increased IL-6 levels and memory and learning performance after 4.5–6 h [59]. Among all results, the injection of LPS did not alter working or executive functions or attention among young healthy volunteers. With higher doses of LPS (2 ng/kg body weight), the results are also controversial. This treatment did not induce any alteration of working memory, psychomotor speed capacity and information processing ability, fine control motor and attention performances until 10 h post-injection compared with the placebo group [60]. Surprisingly, the authors observed an increase in attention performance in the treated group compared to the placebo group.

In 2010, Grigoleit et al. [61] found that the injection of LPS did not affect the subscales of the Wechsler Memory Scale, analysing performances in verbal, visual or delayed memory, as well as attention and executive control processes. Using a double-blind crossover study, the same authors [62] observed that LPS injection did not affect accuracy in working memory performance, but improved reaction time in the high-dose (0.8 ng/kg body weight) 2 h post-injection compared to placebo; a result that was not observed with the lowest LPS dose (0.4 ng/kg body weight).

Alteration in emotional/social processing was observed following the injection of LPS at 0.8 ng/kg body weight [63], but not with a lower dose of LPS (0.4 ng/kg body weight) [64].

Altogether, these results underlined that the LPS dose, the delay and the targeted samples have different responses on the cognitive performances assessed (themselves being heterogeneous between studies), which deserves further research with emphasis on both experimental conditions and outcomes.

#### 3.2.2. Endotoxemia and HIV-Associated Neurocognitive Disorders in Observational Studies

In the field of LPS and cognition, we identified 2 cross-sectional observational studies on non-healthy individuals, focusing on HIV-infected participants with or without HIV-associated neurocognitive disorders (HAND) [65,66] (Table 3). The median fasting levels of LPS were higher among the HAND group than among the no-HAND participants (116.1 ρg/mL vs. 98.2 ρg/mL) [65]. Interestingly, circulating levels of LPS were not associated with the severity of HAND. In the other study, plasma LPS levels did not differ according to the score of a neurocognitive test battery designed to assess several domains of cognitive function (i.e., motor skills, speed of information processing, attention, learning, memory, language fluency and executive function) [66]. More recently, Jespersen et al. [67] reported a lack of association between fasting levels of LPS and markers of axonal damage or monocyte activation in the central nervous system among HIV-infected adults without evidence of impaired cognitive function. In this specific sample, LPS was not detectable in the cerebrospinal fluid.

Although not tested in a human model of endotoxemia, repeated lifelong exposure to endotoxin may lead to a long-term alteration in all cognitive domains. Dementia diagnosis is the result of a long insidious process where cognitive disorders evolve to pathology. However, the relationship between endotoxemia and dementia has been explored in very few studies, which are described below (Table 4).

#### 3.2.3. Endotoxemia and Alzheimer’s Disease or Dementia Diagnosis in Observational Studies

First, we identified two studies interested in the anatomy of the brain in post-mortem patients. Recent investigations reported higher LPS abundance in grey matter (superior temporal gyrus lobe) and white matter (frontal lobe) in brains from patients with Alzheimer’s disease (AD) than in those from participants free of dementia [68]. Of greater interest, LPS colocalized with Aβ1-40/42 in amyloid plaques and with Aβ1-40/42 around blood in AD brains [68]. Another study reported an average three-fold higher abundance of in the hippocampus—an anatomical region of the AD brain that develops the earliest and most profound neuropathology—from four AD brains compared to two age-matched control brains, as well as a two-fold higher abundance in neocortical extracts from six AD brains and six age-matched control brains [69]. In some advanced AD patients (criteria not defined in the study), hippocampal brain lysate exhibited up to a 26-fold increase in LPS [69].

In 2008, Ancuta et al. [70] observed that plasma LPS levels were significantly higher among dementia-associated HIV participants than among participants without neurocognitive impairment, independent of plasma viral load and CD4 counts. Surprisingly, LPS levels in participants with minor cognitive and motor disorders, asymptomatic neurocognitive impairments or neuropsychiatric impairments did not differ from those in participants without neurocognitive impairment. Finally, the last study we identified reported that plasma LPS levels were three-fold higher in 18 AD participants (mean 61 ρg/mL) than in 18 healthy controls (mean 21 ρg/mL) [71].

Altogether, the scarce available literature in this field suggests that higher LPS levels are observed (i) among the brains of AD patients, in several regions of interest and (ii) among living AD participants, and that these results could be partially explained by higher immunosenescence also among HIV patients. These results allow us to speculate that not only transient immune activation induced by LPS, as described earlier, but also increased fasting levels of LPS (from different sources) could be partly involved in the pathogenesis of dementia. Longitudinal studies are still required to test this hypothesis. 

## 4. Discussion

The hypothesis tested in this “state-of-the-art” paper was that metabolic endotoxemia could be an underlying mechanism of the relationship between nutrition (and mainly fats intake) and age-related cognitive impairments.

First, to the best of our knowledge, there was no study focusing on this whole association (i.e., nutritional habits that could modulate endotoxemia, which itself could be part of the pathological processes leading to dementia) in a single sample setting. Therefore, we conducted two approaches, based on the available literature: we synthesized studies focusing (i) on the role of nutritional habits and interventions on the modulation of metabolic endotoxemia, and (ii) on the association between endotoxemia and cognitive impairments.

To some extent, overall metabolic endotoxemia may be attributed to a balance between the number of LPS-containing Gram-negative bacteria in the gut microbiota and the subsequent translocation of the LPS across the gastro-intestinal barrier into the bloodstream; the fat content of the diet being a putative key actor of this translocation.

Our synthesis provided convincing evidence that fat-rich meals have an undoubted capacity to transiently modulate postprandial metabolic endotoxemia in humans. Indeed, recent studies have demonstrated that fat absorption and digestion is a step where dissociated LPS can be incorporated into chylomicrons (i.e., lipoproteins responsible for the transport of lipids through the gut barrier) thereby enabling their translocation into the bloodstream [21,22]. We highlighted that not only the quantity but also the quality of dietary fats may influence metabolic endotoxemia. Precisely, imbalanced diets in favour of saturated fatty acids have been associated with higher postprandial levels of LPS while a combination of these diets with other healthy nutritional components, such as fibre [41], is able to limit this increase.

On the other hand, long-term consumption of a high-energy-density diet, especially those derived from fat, has been associated with gut microbiota dysbiosis [72]. By shifting the balance in favour of, or at the expense of, LPS-containing Gram-negative bacteria, diet could also contribute to the amount of LPS in the gut and their translocation into the bloodstream. Metabolic diseases such as obesity and T2DM are often associated with gut microbiota dysbiosis [73,74], most likely worsened by lifelong consumption of an unhealthy fat-rich diet. This latter result could explain, at least in part, the results reporting increased fasting levels of LPS and an exacerbated postprandial increase of LPS following a high-fat-saturated meal in metabolically impaired individuals compared to healthy individuals.

Moreover, gut dysbiosis, and the subsequent release of LPS can be managed by several dietary factors, that are not discussed in the present paper, including, for instance, the intake of refined sugars, alcohol or nutritional supplements with pre- and probiotics for details see, [19]. In particular, high consumption of glucose or fructose, which is part of the Western diet, could induce an increase in circulating levels of LPS in mice [75]. Pronounced intestinal permeability and increased plasma levels of LPS were found in patients with chronic alcohol abuse [76]. Pre- and probiotics have demonstrated the ability to manipulate gut microbiota and to influence the circulating levels of LPS [19]. However, we did not intend to review all potential and nutritional factors that might increase levels of LPS and prefer to limit our hypothesis to lipids, which is a well-known risk factor for AD [77].

As a limitation of the selected studies, the discrepancies observed between postprandial and fasting levels of circulating LPS after dietary interventions are questionable. These observations suggest that metabolic endotoxemia has a fluctuating nature in humans, and that fasting levels of LPS may therefore not be an accurate marker of chronic exposure to endotoxins over time. Some authors have also suggested the measurement of LBP, which is considered a longer-term marker of endotoxin-related exposure than LPS [78,79].

The main consequence of chronic exposure to endotoxemia is the onset and maintenance of a low-grade inflammation state, with associated deleterious outcomes in elderly individuals [26,27]. However, it remains unclear to what extent acute exposure to LPS could induce age-related cognitive disorders. Indeed, our literature research showed no convincing evidence that exposure to an intravenous overload of endotoxins was associated with major cognitive alterations in healthy individuals; a result that is in line with a previous review [80]. As already mentioned, the large heterogeneity of interventional studies reported in this paper (i.e., in terms of the injected doses of LPS, the neurocognitive tests used to assess short-term cognitive performances or the various delays between injection of LPS and cognitive assessment, for instance) limits us in drawing definitive conclusions regarding the association between endotoxemia and cognitive performances. Additionally, speculative, controversial results of LPS injection on some selected cognitive tests also ask for questions on possible compensatory mechanisms, which deserves further research.

Regarding the association between endotoxemia and dementia, the results seem to be more consistent, while they are based on a very small number of studies. Dementia results in a long insidious process accompanied by molecular and physiological changes, including oxidative stress, impairment in neuronal function and the death of neuronal cells, which may be caused or worsened by neuroinflammation [26,81]. We thought that accumulated lifelong exposure to endotoxin may therefore be associated with more severe stages of cognitive disorders and dementia. Due to the lack of prospective studies to support this hypothesis, we cannot exclude a possible reverse causation. For instance, individuals with advanced stages of dementia are more likely to develop bacterial infections (i.e., in part due to a lack of hygiene inherent to the deterioration of cognitive performances and disability status) and therefore to be exposed to higher endotoxemia. In addition, and inherent to ageing, increased permeability in physiological barriers (i.e., intestinal and blood-brain barriers) is observed in AD individuals [82,83], which could also promote the translocation of higher amounts of neurotoxic molecules such as LPS.

Finally, the LPS doses used in most interventional studies may also be discussed, in addition to those of natural exposure pathways which may gradually and intermittently deliver smaller amounts of endotoxin over time.

Among the limitations of this state-of-the-art paper, we acknowledge the lack of adopting a strict systematic literature review methodology. However, only partial studies have been identified to respond to our whole hypothesis which confirmed our innovative approach. As a limitation, we cannot exclude a publication bias; studies that are not statistically significant have been available more often than those with significant results. The strengths of the present analysis are therefore to update, by a holistic approach, in a single article the experimental and observational literature in the field of fat-rich nutrition, endotoxemia and cognition in humans and to identify some gaps to be completed in the near future.

## 5. Conclusions

Nutrition has been proposed as a promising non-medical strategy to prevent cognitive decline and subsequent dementia. Affected by the quantity and the quality of ingested fats, metabolic endotoxemia, involving a potent pro-inflammatory response of the host, could be one of the underlying mechanisms. As the postprandial state represents a stressful condition in which our current society spends most of its time, the identification of an individual-adapted dietary pattern associated with lower metabolic endotoxemia and subsequent inflammation is a promising area for future research focusing on inflammatory-associated diseases. However, there is an important need for research to understand to what extent transient but also chronic low-exposure to LPS, through repeated measurements of postprandial and fasting levels of LPS over time, could be associated with long-term cognitive changes until the diagnosis of dementia.

## Figures and Tables

**Table 1:** Selective evidence on the association between a single high-fat meal and metabolic endotoxemia.

Clinical Trials					
Reference	Sample Characteristics	Endotoxemia Assessment	Nutritional Characteristics	Analysis Design	Results
Lyte JM. et al., Lipids Health Dis, 2016 [37]	20 healthy subjects (mean age 25 y, 60% men)	Serum LPS quantified by LAL	Four meals (35% fat provided from n-3 PUFA (fish oil), 35% fat provided from n-6 PUFA (grapeseed oil), 35% fat provided from SFA (coconut oil) and 20% fat (olive oil, control diet))	Randomized single-blind crossover study	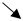 LPS until 5 h after n-3 PUFA meal compared to SFA meal
Al-Disi DA. et al., Nutrients, 2015 [45]	92 lean controls (mean age 24 y), 24 overweight or obese (mean age 32 y) and 50 T2DM (mean age 42 y) subjects, all women	Serum LPS quantified by LAL	High-fat meal (whipping cream with 75 g fat, 5 g carbohydrate and 6 g protein per m² body surface)	Randomized controlled trial	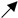 LPS 3 h after high-fat meal in the lean (compared to baseline) and T2DM (compared to 1 h postprandial) groups
Vors C. et al., J Clin Endocrinol Metab, 2015 [36]	8 normal-weight (mean age 29 y) and 8 obese (mean age 31 y) subjects, all men	Plasma LPS quantified by LAL	Mixed meals containing 10 or 40 g fat (69% SFA, 28% MUFA and 3% PUFA)	Randomized crossover study	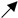 LPS only in obese subjects after 40 g fat compared to 10 g fat
Schwander F. et al., J Nutr, 2014 [35]	19 normal-weight (mean age 41 y) and 18 obese (mean age 44 y) subjects, all men	Plasma LPS quantified by LAL	500, 1000 and 1500 kcal of a high-fat meal (61% fat)	Randomized crossover study	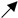 LPS with energy intake in both groups
					No difference in the dose-response of postprandial LPS between normal-weight and obese
Harte AL. et al., Diabetes Care, 2012 [43]	9 non-obese (mean age 40 y, 50% men), 15 obese (mean age 44 y, 50% men), 12 impaired glucose tolerance (IGT, mean age 42 y, 58% men) and 18 T2DM (mean age 45 y, 61% men) subjects	Serum LPS quantified by LAL	High-fat meal (whipping cream with 75 g fat, 5 g carbohydrate and 6 g protein per m² body surface)	Controlled trial	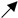 LPS after high-fat diet in all four groups compared to baseline
					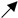 LPS by 20% in obese and IGT and by 125% in T2DM subjects compared to non-obese participants
Milan AM. et al., Nutrients, 2017 [42]	15 young adults (mean age 23 y, 40% men) and 15 elderly subjects (mean age 67 y, 40% men)	Plasma LPS quantified by LAL	High-fat (32% fat) or low-fat (11.5% fat) meals	Randomized crossover study	No difference in LPS levels after high-fat diet compared to baseline
Schmid A. et al., Br J Nutr, 2015 [34]	19 healthy subjects (mean age 42 y, all men)	Plasma LPS quantified by LAL	Three meals (High-fat dairy meal, High-fat non-dairy meal supplemented with milk or not, all 60% fat)	Randomized crossover study	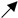 LPS after high-fat meal compared to baseline
					No difference in postprandial LPS levels between the three meals
Moreira AP. et al., J Hum Nutr Diet, 2016 [39]	65 overweight and obese subjects (mean age 27 y, all men)	Plasma LPS quantified by LAL	Three meals (shake with conventional peanuts CVP, high-oleic peanuts HOP or control biscuit CT, all 49% fat)	Controlled trial	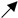 LPS in the CT diet 3 h after ingestion compared to CVP and HOP meals
Clemente-Postigo M. et al., J Lipid Res, 2012 [44]	10 subjects with HOMA-IR ≤ 5 and TG < 80 mg/dL (group 1, mean age 40 y), 10 subjects with HOMA-IR > 8 and TG < 80 mg/dL (group 2, mean age 39 y), 10 subjects with HOMA-IR ≤ 5 and TG > 80 mg/dL (group 3, mean age 43 y) and 10 subjects with HOMA-IR > 8 and TG > 80 mg/dL (group 4, mean age 42 y), all morbidly obese subjects	Serum LPS quantified by LAL	High-fat meal (50% fat)	Controlled trial	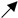 LPS after high-fat meal in groups 3 and 4 (TG > 80 mg/dL) compared to baseline
					No difference in LPS levels after high-fat diet between groups
Deopurkar R. et al., Diabetes Care, 2010 [32]	48 healthy subjects (range age 25–47 y)	Plasma LPS quantified by LAL	33 g dairy cream (70% SFA, no carbohydrate), 75 g glucose-based drink, orange juice or water	Controlled trial	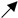 LPS after ingestion of dairy cream compared to baseline
					No difference in LPS levels after ingestion of glucose-based drink, orange juice or water
Ghanim H. et al., Diabetes Care, 2009 [40]	20 healthy subjects (range age 20–50 y, 75% men)	Plasma LPS quantified by LAL	High-fat high cholesterol HFHC (42% fat) or American Heart Association (AHA)-recommended (27% fat)) meals	Controlled trial	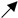 LPS after HFHC meal compared to baseline
					No difference in LPS levels after AHA diet compared to baseline
Ghanim H. et al., J Clin Endocrinol Metab, 2017 [41]	10 healthy subjects (mean age 33 y, 60% men)	Plasma LPS quantified by LAL	High-fat high-carbohydrate HFHC meal (42% fat) with or without additional 30 g of fibre	Randomized crossover study	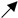 LPS after HFHC meal
					No difference in postprandial LPS levels with additional 30 g of fibre to the HFHC
Erridge C. et al., Am J Clin Nutr, 2007 [33]	12 healthy subjects (mean age 32 y), all men	Plasma LPS quantified by LAL	High-fat meal (toast with 50 g butter)	Randomized crossover study	LPS after high-fat meal
Laugerette F. et al., J Nutr Biochem, 2011 [21]	12 healthy subjects (mean age 27 y, all men)	Plasma LPS quantified by LAL	Mixed meal (33% fat)	Controlled trial	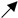 LPS after mixed meal compared to baseline
Vors C. et al., Lipids Health Dis, 2017 [38]	8 normal-weight (mean age 29 y) and 8 obese (mean age 31 y) subjects, all men	Plasma LPS quantified by LAL	Meals with emulsified or spread fat	Randomized crossover study	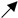 LPS after emulsified fat meal in obese subjects compared to (i) spread fat and (ii) emulsified or spread fat in normal-weight subjects

Abbreviations: LPS Lipopolysaccharide; LAL Limulus Amebocyte Lysate; SFA Saturated fatty acids; MUFA monounsaturated fatty acids; PUFA polyunsaturated fatty acids; T2DM Type 2 Diabetes Mellitus; HOMA-IR Homeostasis model assessment of insulin resistance; TG Triglycerides; IGT impaired glucose tolerance.

**Table 2:** Selective evidence on the association between long-term diet and endotoxemia.

Clinical Trials					
Reference	Sample Characteristics at Baseline	Endotoxemia Assessment	Nutritional Assessment	Analysis Design	Results
Laugerette F. et al., Mol Nutr Food Res, 2014 [46]	18 healthy subjects (mean age 31 y, all men)	Plasma LPS quantified by LAL	Overfeeding with +70 g of lipids to the usual daily diet with 46.3% from saturated fatty acids during 8 weeks	Controlled trial	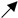 Postprandial levels of LPS compared to baseline
					No difference in fasting levels of LPS after overfeeding period compared to baseline
Breusing N. et al., J Am Coll Nutr, 2017 [50]	15 healthy subjects (age 20–29 y, all men)	Plasma LPS quantified by LAL	Overfeeding (+50% of the energy requirement) during 1 week, caloric restriction (−50% of the energy requirement, 3.5% fat) during 3 weeks and hyper-caloric refeeding (+50% of the energy requirement) with either low- or high-glycemic index diet during 2 weeks	Randomized crossover study	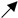 Fasting levels of LPS after overfeeding period (+30.8% compared to baseline)
					Normalization of fasting levels of LPS levels with the caloric restriction diet
					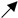 Fasting levels of LPS after hyper-caloric refeeding period (+24.7% compared to baseline)
López-Moreno J. et al., J Agric Food Chem, 2017 [48]	75 subjects with metabolic syndrome (mean age 56 y)	Plasma LPS quantified by LAL	Four diets (High-saturated-fatty acids diet (HSFA, 38% fat with 16% SFA, 12% MUFA and 6% PUFA), High MUFA (HMUFA, 38% fat with 8% SFA, 20% MUFA and 6% PUFA), Low-fat high complex carbohydrate (LFHCC, 28% fat) and LFHCC n-3 supplemented with n-3 PUFA) during 12 weeks	Randomized controlled trial	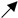 Postprandial levels of LPS after HSFA diet compared to baseline
					No difference in postprandial LPS levels after HMUFA, LFHCC and LFHCC n-3 diets
					No difference in fasting levels of LPS between all 4 groups of diet after intervention
López-Moreno J. et al., Exp Gerontol, 2018 [47]	20 healthy subjects (mean age 67 y, 50% men)	Plasma LPS quantified by LAL	Mediterranean diet enriched in MUFA with virgin olive oil (38% fat) or SFA-rich diet (38% fat) or low-fat high-carbohydrate diet enriched in n-3 PUFA (CHO-PUFA diet, 30% fat) during 3 weeks	Randomized crossover study	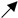 Postprandial levels of LPS after CHO-PUFA
					No difference in postprandial levels of LPS after Mediterranean diets enriched in MUFA or SFA
					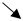 Fasting levels of LPS after CHO-PUFA diet compared to Mediterranean diets enriched in MUFA or SFA
Pendyala S. et al., Gastroenterology, 2012 [49]	8 healthy subjects (mean age 60 y, 38% men) hospitalized for the study	Plasma LPS quantified by a neutrophil priming method	Western-type diet (40% fat with 20.8% from saturated fat, 20% protein and 40% carbohydrates) or Prudent-type diet (20% with 5.8% from saturated fat, 20% protein and 60% carbohydrates) during 1 month	Randomized crossover study	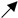 Fasting levels of LPS after Western-type diet (+71% compared to baseline)
					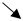 Fasting levels of LPS after prudent-type diet (−38% compared to baseline)
**Observational studies**					
**Reference**	**Sample Characteristics at Baseline**	**Endotoxemia Assessment**	**Nutritional Assessment**	**Analysis Design**	**Results**
Amar J. et al., Am J Clin Nutr, 2008 [51]	130 subjects below the LPS detection threshold (mean age 55 y), 44 subjects between 9–39 U/mL (mean age 54 y) and 27 subjects under 39 U/mL (mean age 53 y), all healthy men	Plasma LPS quantified by Kinetic-QCL ^TM^ test	3 days of food-record diary	Cross-sectional	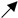 Fasting levels of LPS with fat and total energy intake
Kallio KA. et al., Acta Diabetol, 2015 [52]	2452 subjects (mean age 52 y)	Serum LPS quantified by LAL	24 h dietary recall	Cross-sectional	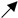 Fasting levels of LPS with total energy intake in lean healthy subjects
					No significant association between fasting levels of LPS and fat intake, and among subjects with obesity, metabolic syndrome, diabetes or coronary heart disease
Röytiö H. et al., Br J Nutr, 2017 [53]	88 overweight pregnant women (mean age 30 y)	Serum LPS quantified by LAL	Three groups based on 3 days of food-record diary (low fibre (<25 g/j) and moderate fat intake (25–40%) n = 57, high fibre (>=25 g/j) and moderate fat intake (25–40%) n=18 and low fibre (<25 g/j) and high fat intake (>=40%) n = 13)	Cross-sectional	No significant association between fasting levels of LPS and fat intake
					No difference in fasting levels of LPS levels among the three diet groups
Ahola AJ. Et al., Sci Rep, 2017 [54]	668 patients with type 1 diabetes (mean age 45 y, 44% men)	Serum LPS quantified by LAL	Food frequency questionnaire and3 days of food-records diary	Cross-sectional	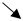 Fasting levels of LPS with higher adherence score of fish, healthy snack and modern diets
					No difference in fasting levels of LPS levels for sweet, cheese, vegetable or traditional diets
					No difference in fasting levels of LPS levels for energy, macronutrients and fibre intake
Pastori D. et al., J Am Heart Assoc, 2017 [55]	704 patients with nonvalvular atrial fibrillation treated by vitamin K antagonists (mean age 74 y, 57% men)	Serum LPS quantified by ELISA	Short food frequency questionnaire	Cross-sectional	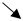 Fasting levels of LPS with higher adherence to a Mediterranean diet
					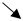 Fasting levels of LPS with higher consumption of fruits and legumes
					No difference in fasting levels of LPS levels for the consumption of olive oil, vegetables, fish, wine, meat and bread

Abbreviations: AD Alzheimer’s Disease; LPS Lipopolysaccharide; LAL Limulus Amebocyte Lysate; SFA Saturated fatty acids, MUFA monounsaturated fatty acids, PUFA polyunsaturated fatty acids.

**Table 3:** Selective evidence on the association between lipopolysaccharides and cognitive function.

Clinical Trials					
Reference	Sample Characteristics at Baseline	Endotoxemia Assessment	Outcomes	Study Design	Results
Krabbe KS. et al., Brain Behav Immun, 2005 [59]	12 healthy subjects (mean age 26 y, all men)	Injection of LPS *E. coli* 0.2 ng/kg or placebo	Memory and learning (Word-list memory test) Working memory (Digit Span backward and Letter–Number Sequencing) Attention (Digit span forward and Digit symbol) Executive functions (Trail Making Test A and B) Assessment before and at 1.5, 6 and 24 h post-injection	Randomized double-blind crossover study	Negative correlation between IL-6 at 4.5 and 6 h post-injection and the word-list learning performance LPS injection did not affect performance on other cognitive tests
Kullmann JS et al., Soc Cogn Affect Neurosci, 2014 [64]	18 healthy subjects (mean age 26 y, all men)	Injection of LPS *E. coli* 0.4 ng/kg or placebo	Emotional/social processing (Reading the mind in the eye) Brain activation during the test Assessment at 2 h post-injection	Randomized double-blind crossover study	LPS injection did not affect the number of correct responses during the Reading the mind in the eye test 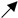 Responses in the fusiform gyrus, temporo-parietal junction, superior temporal gyrus and precuneus regions compared to placebo
Grigoleit JS. et al., Neurobiol Learn Mem, 2010 [61]	24 healthy subjects (mean age 25 y, all men)	Injection of LPS *E. coli* 0.4 ng/kg or placebo	Attention and executive functions (Color word stroop task (assessment before and 1.5 h post-injection)) Verbal, visual and delayed memory (Revised Wechsler Memory Scale (assessment at 3 h post-injection))	Randomized double-blind controlled trial	LPS injection did not affect performance on cognitive tests
Grigoleit JS. et al., PLoS One, 2011 [62]	18 healthy subjects in the low-dose group (LPS 0.4 ng/kg) and 16 subjects in the high-dose group (LPS 0.8 ng/kg), mean age 25 y	Injection of LPS *E. coli* 0.4 ng/kg or placebo, or LPS *E. coli* 0.8 ng/kg or placebo	Working memory (N-back task) Assessment at 1.75 and 3 h post-injection	Randomized double-blind crossover study	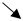 Reaction time in N-back task in the high-dose group compared to placebo LPS did not affect the accuracy in N-back task
Moieni M et al., Brain Behav Immun, 2015 [63]	109 healthy subjects (mean age 24 y, 40% men): 58 subjects in the LPS injection group and 51 in the placebo group	Injection of LPS *E. coli* 0.8 ng/kg or placebo	Emotional/social processing (Reading the mind in the eye) Assessment at 1 h 40 post-injection	Randomized double-blind controlled trial	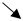 Number of correct responses in the LPS group
Reichenberg A. et al., Arch Gen Psychiatry, 2001 [57]	20 healthy subjects (mean age 24 y, all men)	Injection of LPS Salmonella abortus equi 0.8 ng/kg or placebo	Declarative memory (Story recall, Figure recall and Word-list learning) Attention (Digit span forward, Digit symbol, Ruff 2 and 7 cancellation test, Simple reaction time test and Continuous performance test) Executive functions (Colored Trail Making Test A and B and Word fluency test) Assessment at 1–2, 3–4 and 9–10 h post-injection	Randomized double-blind crossover study	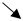 Performance on story recall, figure recall and word-list learning tests compared to placebo at 1–2, 3–4 and 9–10 h post-injection LPS injection did not affect performance on other cognitive tests
Cohen O. et al., J Mol Neurosci, 2003 [58]	10 healthy subjects (sub-sample of the Reichenberg’s study [57])	Injection of LPS Salmonella abortus equi 0.8 ng/kg or placebo	Declarative memory (Story recall) Working memory (Digit span backward) Attention (Digit span forward and Ruff 2 and 7 cancellation test) Assessment at 1–2, 3–4 and 9–10 h post-injection	Randomized double-blind crossover study	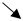 Performance on story recall compared to placebo at 1–2, 3–4 and 9–10 h post-injection 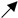 Performance digit span backward test compared to placebo at 1–2, 3–4 and 9–10 h post-injection LPS injection did not affect performance on other cognitive tests
Van den Boogaard M. et al., Crit Care, 2010 [60]	15 healthy subjects in the injection group (mean age 23 y, all men) and 10 healthy controls (mean age 25 y, all men)	Injection of LPS *E. coli* 2 ng/kg or no injection	Working memory (Digit span backward) Attention (Digit span forward, Color word stroop task and Paced Auditory Serial Addition Test (PASAT)) Psychomotor speed capacity and information processing ability (number of correct response on the Digit symbol test) Fine control motor (time required to finish the Grooved pegboard test) Assessment at 1–2, 3–4 and 9–10 h post-injection	Single-blind trial	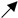 Performance on PASAT test compared to control groupLPS injection did not affect performance on other cognitive tests
**Observational studies**					
**Reference**	**Sample characteristics at baseline**	**Endotoxemia assessment**	**Outcomes**	**Study design**	**Results**
Lyons JL. et al., J Acquir Immune Defic Syndr, 2011 [66]	97 HIV-infected patients (mean age 47 y, 76% men)	Plasma LPS quantified by LAL	HIV-associated neurocognitive disorders (HAND) Global T test (motor skills, speed of information processing, attention (working memory), learning (memory encoding), memory (memory recall), language fluency, and executive function)	Cross-sectional	LPS levels did not differ according to the severity of HAND LPS levels did not differ in subjects with global impairment (Global T test < 40) compared to unimpaired subjects
Vassallo M. et al., J Neurovirol, 2013 [65]	40 HIV-infected patients with HIV-associated neurocognitive disorders (HAND) (median age 46 y, 78% men) and 139 HIV-infected patients without HAND (median age 44 y, 72% men)	Plasma LPS quantified by LAL	HIV-associated neurocognitive disorders (HAND) z-score (learning and recall episodic memory, attention/concentration, working memory, executive functions, language, visual agnosia and motor/psychomotor speed)	Cross-sectional	LPS levels was higher in the HAND group compared to no-HAND group LPS levels did not differ according to the severity of HAND
Jespersen S. et al., BMC Infect Dis, 2016 [67]	62 untreated HIV-infected patients without evidence of impaired cognitive function (mean age 39 y, 52% men)	Plasma and CSF LPS quantified by LAL	CSF neurofilament light chain protein (marker of CNS axonal damage) and CSF neopterin (marker of monocyte activation)	Cross-sectional	No association between plasma LPS and CSF neurofilament light chain protein or CSF neopterin LPS was not detectable in CSF

Abbreviations: AD Alzheimer’s Disease; LPS Lipopolysaccharide; LAL Limulus Amebocyte Lysate; HIV Human Immunodeficiency Viruses; HAND HIV-associated neurocognitive disorders; CSF Cerebrospinal Fluid; CNS Central Nervous System; NFL Neurofilament light chain protein.

**Table 4:** Selective evidence on the association between lipopolysaccharides and dementia.

Alzheimer’s Disease Brain Samples					
Reference	Sample Characteristics at Baseline	Endotoxemia Assessment	Outcomes	Study Design	Results
Zhan X. et al., Neurology, 2016 [68]	24 AD brains (mean age 77 y, 38% men, median Braak stage: 6) and 18 age-matched controls brains (mean age 81 y, 56% men, median Braak stage: 2)	LPS *E. coli* antibodies and *E. coli* K99 pili protein	Superior temporal gyrus from grey matter (GM) and frontal lobe from white matter (WM)	Cross-sectional	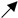 Detection of *E. coli* K99 and LPS levels in GM and WM AD compared to control LPS colocalized with Ab1-40/42 in amyloid plaques and with Ab1-40/42 around blood in AD brains
Zhao Y. et al., Front Cell Infect Microbiol, 2017 [69]	10 AD brains (mean age 74 y) and 8 controls brains (mean age 73 y), all women	LPS *E. coli* antibodies	Neocortex (temporal lobe) and hippocampus	Cross-sectional	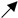 LPS on average over two-fold in AD neocortex compared to controls 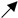 LPS on average over three-fold in AD hippocampus compared to controls (up to 26-fold in some advanced AD)
**Alzheimer’s disease and dementia diagnosis**					
Zhang R. et al., J Neuroimmunol, 2009 [71]	18 AD patients (mean age 79 y, 39% men) and 18 healthy controls (mean age 55 y, 67% men)	Plasma LPS quantified by LAL	Alzheimer’s disease	Cross-sectional	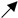 LPS in AD patients (61 ρg/mL on average) compared to healthy controls (21 ρg/mL on average)
Ancuta P. et al., PLoS One, 2008 [70]	119 HIV patients whose 32 with No-NCI, 28 with HIV-associated dementia, 25 with MCMD, 20 with NPI-O, 9 ANI and 5 unable to assign	Plasma LPS quantified by LAL	HIV-associated dementia (HAD, defined as the involvement of at least two cognitive domains and documented by a performance of two SD below the normative mean on neuropsychological tests, with marked interference in daily functioning)	Cross-sectional	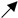 LPS in HAD compared to No-NCI HIV-patients LPS levels did not differ in MCMD, NPI-O or ANI compared to No-NCI patients

Abbreviations: AD Alzheimer’s Disease; LPS Lipopolysaccharide; LAL Limulus Amebocyte Lysate; HIV Human Immunodeficiency Viruses.
